# A sustainable methodological approach for mitigation of salt stress of rice seedlings in coastal regions: Identification of halotolerant rhizobacteria from Noakhali, Bangladesh and their impact

**DOI:** 10.1016/j.mex.2024.102981

**Published:** 2024-10-01

**Authors:** F.M. Ashik Mahmud, Md. Aminul Islam, Mehede Hassan Rubel, Prosun Bhattacharya, Firoz Ahmed

**Affiliations:** a Department of Microbiology, Noakhali Science and Technology University, Noakhali 3814, Bangladesh; b COVID-19 Diagnostic Lab, Department of Microbiology, Noakhali Science and Technology University, Noakhali 3814, Bangladesh; c Advanced Molecular Lab, Department of Microbiology, President Abdul Hamid Medical College, Kishoreganj, Bangladesh; d Department of Agriculture, Noakhali Science and Technology University, Noakhali 3814, Bangladesh; e Department of Sustainable Development, Environmental Science and Engineering, KTH Royal Institute of Technology, Teknikringen 10B, Stockholm SE 10044, Sweden

**Keywords:** Salt stress, HPGPR, Bioinoculant, Rhizobacterial effects, Identification of halotolerant rhizobacteria and their impact on rice seedlings in salt stress

## Abstract

Salinity hinders the growth of many crops common in the diet, such as rice, wheat and maize when cultivated in coastal salinity areas. Given the limited availability of cultivable land and the increasing growth of the population, it is necessary to enhance productivity. In this paper, we present an innovative approach to adopting Halotolerant Plant Growth Promoting Rhizobacteria (HPGPR) to enhance salt-tolerant rice varieties to solve salinity stress and enhance crop production. HPGPR has functions to overcome plant growth and development and is the most efficient bioinoculant for rice in saline environments. This approach can be considered a potential method because of the cost-effective and environmentally friendly impacts in agricultural production, which involves salt-affected areas.

## Graphical abstract

**Figure:**
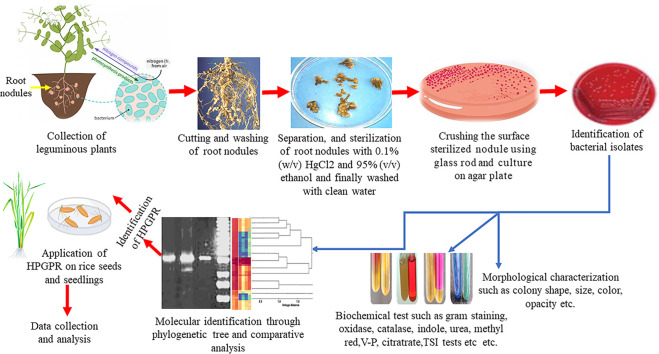

Specifications table

**Table:** 

Subject area:	Microbiology
More specific subject area:	Agricultural microbiology
Name of your method:	Identification of halotolerant rhizobacteria and their impact on rice seedlings in salt stress
Name and reference of original method:	Effects of halotolerant rhizobacteria on rice seedlings under salinity stress. (https://doi.org/10.1016/j.scitotenv.2023.163774)
Resource availability:	Original research article

## Background

Salinity poses a significant problem to agricultural cultivation in coastal zones. Drought, salinity, severe temperatures, and other biotic and abiotic causes have devastated the earth's surface [5]. The world's population is increasing and the area of arable land is reducing; new approaches are required to increase yields in salts. Among most potential approaches, Halotolerant Plant Growth Promoting Rhizobacteria (HPGPR) has been identified as a highly effective solution. These bacteria are adapted to saline environments, fix nitrogen from the soil and provide nutrition to the plants through the production of plant hormones, conversion of insoluble phosphates into soluble forms, and enhancement of nutrient adsorption. They are most crucial in converting insoluble phosphates in the soil into forms plants can use, which are fundamental in energy transfer, photosynthesis amongst others [4,14,16]. The objective of this research is to isolate HPGPR from root nodules of legume plants and assess its ability to enhance the growth of rice plants under salinity stress conditions. With these newly found HPGPR strains, it is our aim to enhance plant growth in saline stress and to facilitate and encourage the use of bio-fertilizers from this source.

Old practices of altering soil properties and implementing efficient irrigation systems are expensive and practically unachievable for smallholder farmers. While chemical fertilizers cause environmental issues and health complications, bio-fertilizers provide a more economical and ecological solution to increase fertility of the soil and the health of the plants by introducing useful microorganisms. The combination of salt-tolerant rice cultivars with HPGPR offers the possibility of combating salinity stress as a two-pronged strategy. This two-in-one model holds great promise for boosting crop production in areas affected by salinity, securing food production and agriculture in the process. This study shall give a practical application approach to utilizing these rhizobacteria in agricultural production in coastal lands.

## Method details

**Bacterial identification:** Legume plants were harvested from several Bangladeshi coastal locales where nodules on neighboring roots were present, including yard-long beans, common beans, shame plants, and dhaincha. The sites exhibited a range of soil textures, transitioning from sandy loam to sandy clay loam. The soil pH levels varied between 7.83 and 8.10, while the highest recorded soil organic matter content was 6.22 % (Table 1). Afterward, the loose roots underwent a gentle rinsing with tap water, followed by a 5-minute sterilization in a 0.1 % (w/v) solution of mercuric chloride (HgCl2). Subsequently, a sterilization step of 10 s in 95 % (v/v) ethanol was conducted, and the roots were ultimately washed with sterile distilled water. An opaque-white suspension resulted from crushing them with a glass rod that had undergone sterilization to release bacteria into 5 mL of sterile distilled water. The spread plate method was employed to apply a 0.1 mL aliquot of the suspension onto an autoclaved YEMA (Yeast Extract Mannitol Agar) plate [9]. After incubating for 3–4 days at 29 °C, the plates were streaked again onto YEMA medium to obtain a pure culture. Finally, halotolerant rhizobacterial culture stock, single colonies from pure cultures were kept in −80 °C for additional further research.

**Table 1:** Collection sites description, soil characteristics and growth stages for durum wheat samples used in this study.

Location	Collection Date	Location characteristics			Soil characteristics				Growth Stage
		Latitude	Longitude	Elevation (m)	Soil Texture	EC (dS/m)	pH	OM%	
NSTU campus	15th March 2021	90.8925° E to 91.3782°E	22.4534° N to 23.1327° N	−380	Sandy loam	3.320	7.830	2.970	Flowering stages
	21st 01 April 2021			−373	Sandy loam	17.700	7.870	6.220	
	15th April 2021			−249	Sandy clay loam	6.490	8.100	1.410	

### Morphological characterization

The bacterial isolates were analyzed for their colony morphology on YEMA, TY (Tryptone Yeast extract), and MacConkey agar plates, following the procedures outlined by [1]. Following an incubation period of 3 to 4 days at 29 °C, the colonies were examined individually and classified based on their distinct features, including shape, size, color, and other observable traits (Table 2). Various strains of bacteria were subjected to salt tolerance testing by inoculating them on TY agar media containing different concentrations of NaCl: 1 %, 2 %, 3 %, 3.5 %, and 4 % (w/v). Additionally, different bacterial isolates were cultured in media with pH values ranging from 4.0 to 11.0 by using HCl and/or NaOH to ascertain their tolerance to these ranges [6]. Colony formation was examined to determine viability after all incubated plates at various temperatures, including 38°, 40°, 43°, and 45 °C [10].

**Table 2:** Summary of colony morphology, cultural, biochemical and molecular (16S rRNA gene) identification of potentially halotolerant rhizobacteria that were isolated from several nodules on the roots of legume plants.

	Colony morphology						Biochemical tests									Molecular identification				
Isolates	Source of root nodules	Growth media	Colony shape	Colony size	Opacity	Colony colour	Gram staining	Oxidase	Catalase	Indole	Urea	Methyl red	Voges Proskauer	Citrate	Triple Sugar Iron	Related bacterial strain	Identity (%)	Bacterial strains (In this study)	Gene bank accession (In this study)	Published Gene bank accession
Isolate-1	Common Bean (C.B)	YEMA	Circular/ Round	Small	Translucent	Watery white	- ve, rod	+	+	–	+	+	+	–	H_2_S gas (–ve), Fermentation (-ve)	*Agrobacterium tumefaciens* strain CN3	100 %	Agrobacterium saline tolerance strain NSTU1	OM348542	OK355396.1
Isolate-6	Yard long Bean (Y.B)	YEMA	Circular	Small	Opaque	Milky white	+ ve, rod	+	+	–	–	+	+	+	H_2_S gas (–ve), Fermentation (+ve)	*Bacillus subtilis* strain CLW-BA1	98.55 %	Bacillus sp. (in: Bacteria) strain NSTU3	OM365391	HQ334986.1
Isolate-14	Shameplants (Mimo)	YEMA	Circular	Small	Opaque	White	+ ve, rod	+	+	–	–	+	+	–	H_2_S gas (–ve), Fermentation (-ve)	*Lysinibacillus fusiformis* strain 4	98.32 %	Lysinibacillus sp. strain NSTU4	OM365427	MN911368.1

**Legends**: +: indicates positive and -: indicates negative.

### Biochemical characterization

Based on morphological observation, biochemical analysis was conducted for suspected rhizobacterial isolates more precisely. Several microbiological tests were conducted, including gram staining, catalase testing, oxidase testing, MR-VP (Methyl Red-Voges Proskauer) testing, MIU (Motility, Indole, Urease) testing, citrate utilization testing, and triple sugar iron (TSI) testing. Using standard gram staining techniques, the morphological characteristics (cell shapes) of the rhizobacterial isolates were assessed under a microscope. Pink or red colors were used to identify gram-negative bacteria, and purple or blue colors were used to identify gram-positive bacteria (Table 2) [18].

To perform oxidase test, a filter paper strip was dipped into a freshly produced Kovacs Oxidase Reagent. A positive test result was indicated by the appearance of an intense purple color on the strip paper after a small amount of culture was applied to the paper with a sterile loop [13]. In order to assess catalase activity, a modest quantity of bacterial culture was placed on a dry, sterile glass slide, followed by the application of a few drops of 3 % H_2_O_2_ (catalase reagent) on it (Table 2). This allowed to identify the presence or absence of foam or bubbles to be studied [15].

The capacity of an organism to utilize citrate as its exclusive energy source was assessed using Simmon's agar slants, while citrate-negative organisms do not use, those that are citrate-positive cause the medium to turn blue. On the other hand, urease and indole production were assessed using MIU medium. When the color changes from yellow-orange to pink-red, the urease test is positive. No color change indicates a negative organism. The indole test is affirmative if, after adding Kovac's reagent, a pink-red color ring on top appears in the test tube; otherwise, it is negative. There was a 24-hour incubation period at 37 °C for both the Simmon's agar slants and the MIU medium [3].

The bacterial broth was supplemented with MR-VP broth, which is composed of peptone, dextrose, and potassium phosphate. The resulting mixture was then subjected to incubation at 37 °C for a duration of 48 h to determine the result. After that the test tube had been incubated to perform the MR tests. Then, 3–5 drops of methyl red, a pH indicator, were added and a permanent red hue is left behind on the medium's surface indicating positive MR test, whereas a yellow tint is left behind by negative ones. To conduct a VP assay, 3 drops of 5 % alpha-napthol solution (Barritt's-A reagent) and 1 drop of 40 % potassium hydroxide solution (Barritt's-2/B reagent) were added to the bacterial broth, and the broth was subjected to 48 h of incubation at 37 °C while being vigorously agitated. Positive VP tests were indicated by a pink-red, ruby pink, or crimson hue appearing 15 min to 1 hour later, while negative VP tests were indicated by a copper-like or yellow hue appearing (Table 2) [11].

The TSI test was performed to evaluate the potential for hydrogen sulphide production and the capacity of carbohydrate fermentation (glucose, sucrose and lactose). The possibility of TSI medium, which was inoculated with bacteria in a test tube, fermenting a carbohydrate substrate for acid production was tested by incubating at 35–37 °C in air for 18–24 h. After incubation, the presence of a red slant and yellow butt, or a yellow slant and yellow butt, indicating a positive fermentation test, while a red slant and red butt, indicate no carbohydrate fermentation. At the same time, if H_2_S is produced, the black colour of ferrous sulphide was seen in the medium, and bubbles or cracks in the agar indicating the production of H_2_S gas (Table 2) [12].

### Molecular characterization

Three bacterial isolates were selected for molecular analysis (Partial 16S rRNA Gene Sequence) based on morphological and biochemical analysis. Genomic DNA from certain strains of bacteria was extracted using the protocol outlined in the Genomic DNA Kit (InvitrogenTM PureLinkTM Pro 96) [7]. Utilizing a NanoDrop™8000 spectrophotometer, a state-of-the-art instrument manufactured by Thermo Scientific in California, United States, the DNA extractions were meticulously scrutinized for their concentration. Subsequently, to amplify the 16S rRNA gene, polymerase chain reaction (PCR) was performed on a Thermal cycler (model Aeris™ 96 wells, manufactured by Esco Micro Pte. Ltd. in Singapore). The detection of salt-resistant bacteria is facilitated through the utilization of universal primer sets, which consist of a forward primer denoted as 27F (5′-AGAGTTTGATCCTGGCTCAG-3′) and a reverse primer denoted as 1392R (5′-GGTTACCTTGTTACGACTT-3′). During the polymerase chain reaction (PCR), the denaturation step was performed at 94 °C for 5 min, followed by 35 cycles of annealing at 55 °C for 1 min and 30 s, and extension at 72 °C for 1 min and 30 s. After purifying the extracted DNA products using mini columns for gel band purification (Invitrogen™ PureLink™ gel band purification kit) following the manufacturer's instructions, the specimens were transferred to the Genome Centre situated at Jashore University of Science and Technology in Bangladesh, where DNA sequencing was conducted using automated sequencing technology.

**Fig. 1:**
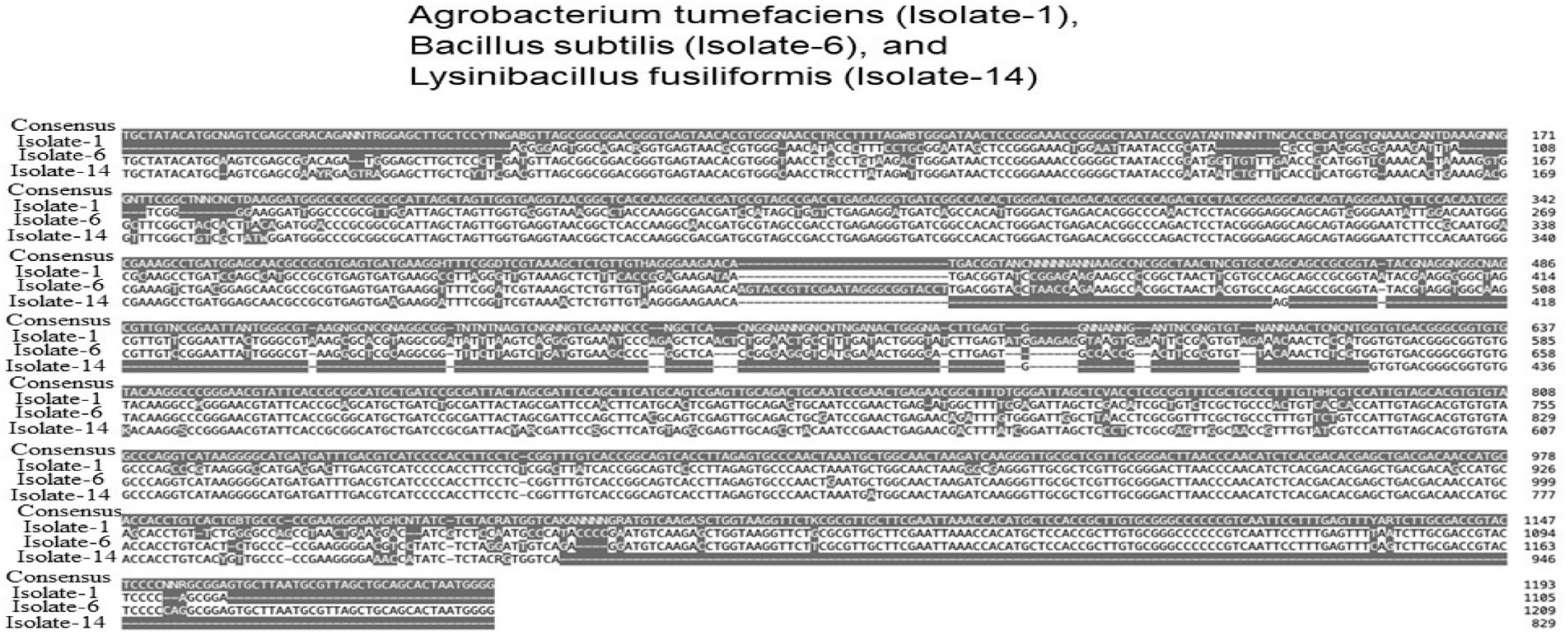
Multiple sequence alignment of 3 isolates namely; *Agrobacterium tumefaciens* (Isolate-1), *Bacillus subtilis* (Isolate-6), and *Lysinibacillus fusiformis* (Isolate-14) rhizobial bacterial sequences using MEGA 5.2 software package showing each bacterial nucleotide sequences.

To compare partial sequences of the 16S rRNA gene from three isolates, the Gene Bank database available at the National Center for Biotechnology Information (NCBI) was employed, utilizing the basic local alignment search tool (BLAST) (Fig. 1; Table 2). The Clustal W program, developed by [17], was utilized to align the matching sequences of the 16S rRNA gene, enabling the identification and analysis of sequence polymorphism. The study encompassed aligning reference sequences, constructing a phylogenetic tree using the Neighbor-Joining method in MEGA 5.2 software, and incorporating bootstrap values derived from 1000 re-samplings to enhance the tree's robustness.

Through molecular identification utilizing phylogenetic tree analysis and comparative analysis, the conclusive findings indicated that Isolate 1 was identified as *Agrobacterium tumefaciens*, Isolate 6 as *Bacillus subtilis*, and Isolate 14 as *Lysinibacillus fusiformis*.

## Method validation

Validation is a vital stage for the preparation of bioinoculant so that their in-vitro effects on crop productivity could be assessed. The ways are followed to identify the most effective bacteria for the sustainable crop production.

### Germination assay

Seeds go through four stages of germination: absorption or imbibition, activation, respiration, and germination. First, water helps to activate the seeds through imbibition; this involves the absorption of water by a seed which helps in absorbing the water and cause cracking of the seed coat. This makes enzymes to discharge nutrient holding capacity, which in convert are utilized in feeding the embryo as it develops. The biochemical rate and cellular respiration to supply energy for the growth of the radicle, which is part of the embryo that would develop into the roots and for the shoot or plumule, which will be the above ground part of the plant. The Binadhan-10 cultivar, a variety of Oryza sativa L., was acquired from the Subornochar substation, a facility of the Bangladesh Institute of Nuclear Agriculture (BINA), situated in Noakhali, Bangladesh. Firstly, collected rice seeds were surface sterilized for five minutes with 3 % sodium hypochlorite (NaOCl) and then, the seeds were soaked in 70 % ethanol for 30 min before being rinsed with distilled water two or three times and dried using a Laminar Air Flow (LAF) hood's filtered air. Following the initial step, separate bacterial suspensions of each isolate (at a concentration of 1 × 10^8^ CFUmL^−1^) were applied as seed drenches onto previously sterilized seeds, which were then subjected to an additional hour of incubation with moderate intensity shaking in the incubator [8]. Subsequently, the seeds were deposited onto a sterilized petri dish that was lined with filter paper and left to desiccate under a laminar air flow hood, with a 14–20 ratio of seeds to plates. Each filter paper, containing seeds inoculated with bacteria or not, was treated with either 3 mL of sterilized distilled water, 1 % NaCl solution, or 1.5 % NaCl solution, ensuring that all bacteria-treated or non-treated seeds received each type of treatment [2]. Upon implementing the designated treatments, encompassing both non-inoculated (untreated) and inoculated (treated) conditions with three diverse bacterial suspensions, alongside treated and untreated variations with NaCl, meticulous germination assays were carried out. Upon placement of the seeds in Petri plates, they underwent a 3-day incubation period in a dark environment at 25 °C, followed by transfer to room temperature for the subsequent germination assay. Germination rates were measured at 48, 72, and 96 h following respective incubation period. The overall length of the shoots, as well as the fresh and dried weight, were measured for this investigation of morphological characteristics. The germination percentage was calculated using the preceding formulas [2].

Germination(%)=(n/N)×100

where ``n'' represents the number of seeds that successfully germinated, and ``N'' represents the total number of seeds under consideration.

Extensive research has confirmed that these three rhizobacteria types significantly enhance plant growth and development, resulting in improved germination rates in various aquatic conditions, including saline and non-saline environments. It's suggesting that these possible HPGPR candidates have potential mitigating effects on salinity stress.

### Pot experiments

**Fig. 2:**
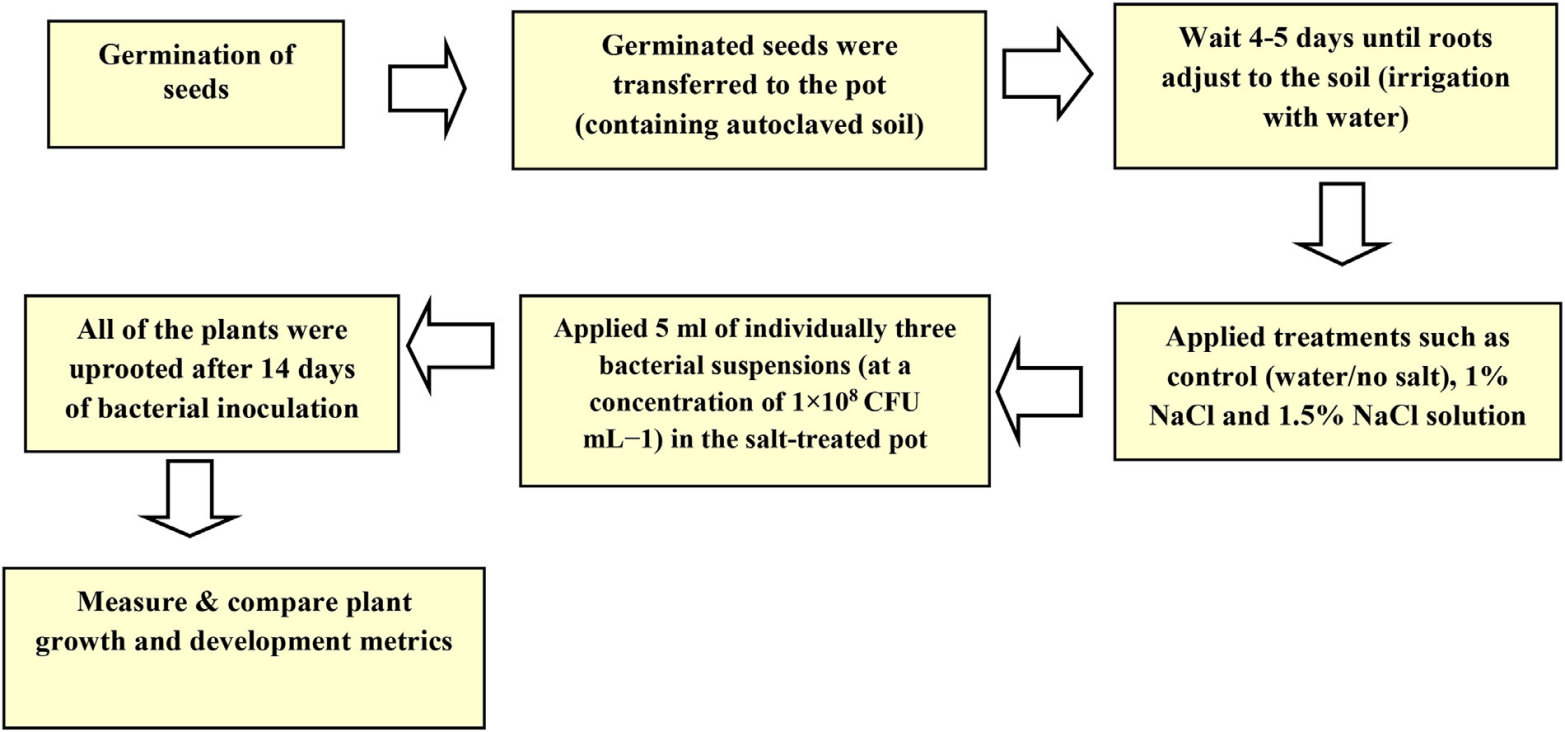
Experimental diagram of pot experiments.

The evaluation of salt tolerance activity exhibited by the aforementioned three bacterial isolates was conducted on rice seedlings that had reached a developmental stage of 14 days. Consistently, sterile seedlings were able to germinate, were then placed into sterilized pots filled with autoclaved soil, and then raised under 26 °C, a light/dark cycle of 16/8 h, and a brightness level of 350 mol m^2^s^-1^. Three distinct salt concentrations, including a control group with no salt, salt administration at 1 % and 1.5 % NaCl, and bacterial application utilizing 5 mL of every bacterial culture (where, each strain had a concentration of 10^8^ colony forming units per mL). To study the interactive effects of NaCl and bacterial isolate treatments on plant growth, the seedlings were subjected to both treatments. Seven rice seedlings were planted in each container, and this process was carried out three times in total. In vitro plant development, the effectiveness of bacterial inoculation under salty conditions was assessed 14-days after inoculation by taking all of the plants and measuring their root and shoot length, fresh weight, dry biomass, total lateral roots, and total chlorophyll content (Fig. 2).

Under saline stress (treated with 1 % and 1.5 % NaCl), rice seedlings inoculated with halotolerant bacteria significantly increased their biomass, growth, and chlorophyll, total length, fresh weight, and dry weight, thereby demonstrating successful adaptation and thriving in high salinity conditions compared to non-inoculated bacteria.

In this study, a total sixteen bacterial isolates from root nodules of leguminous plant were screened based on morphological and biochemical parameters. They were tested for tolerance to temperature (up to 38 °C), salt (up to 3.5 %), and pH (tolerant to pH 11). After that, three isolates were selected for 16S rRNA gene sequencing. A comparison of the 16S rRNA gene sequences revealed that Isolate 1 is similar to *Agrobacterium tumefaciens*, while isolates 6 & 14 are similar to *Bacillus subtilis* and *Lysinibacillus fusiformis* respectively (Table 2, Fig. 1). The plant growth-promoting activities of these bacteria were evaluated by seed germination bioassay (such as measuring germination percentage, total length, and fresh and dry weight) as well as pot experiments to determine (such as root and shoot length, fresh weight, dry biomass, total lateral roots, and chlorophyll content) their potential to favour rice seedling emergence under saline stress. The halotolerant rhizobacteria may have adapted by changing the chemistry of proteins within their own cells to survive in environments with high levels of salt and could be used as bio-fertilizer for mitigating the stress of salinity in salt-sensitive crops. Future research will utilise these bacteria as biofertilizers in salt-affected coastal agricultural areas and explore their molecular-level interactions with host plants.

## Limitations

Not applicable.

## Ethics statements

The authors have all ethical responsibilities of this manuscript. The authors also declared no conflict of interest and none of the animal and/or human trials were included.

## CRediT author statement

**Firoz Ahmed:** Conceptualization of the study. **F.M. Ashik Mahmud**: Preparation of the manuscript drafts. **Mehede Hassan Rubel**: Supervision and edited the manuscript. **Md. Aminul Islam**: Writing original manuscript, draft finilaziation, review and addressed reviewer questions, conducted partial sequencing with sequence submission to NCBI. **Prosun Bhattacharya:** Reviewing and editing. All authors read and approved the final draft of the manuscript.

## Isolation and identification

Sample collection from root nodules from coastal regions, isolate and characterise salt-tolerant rhizobacteria.

## Application and assessment

HGPR in combination with salt-tolerant rice cultivars will be further subjected to field trials in order to assess the impact of rhizobacteria on plant growth.

This study shows that there is the possibility of HPGPR increasing plant growth attributes, that can be used as biofertilizers. This methodology could at least give an insight as to the present microbial population and provide effective biofertilizers for saline areas.

## Declaration of competing interest

The authors declare that they have no known competing financial interests or personal relationships that could have appeared to influence the work reported in this paper.

## Data Availability

Data will be made available on request.

## References

[1] Beringer J.E. (1974). R factor transfer in Rhizobium leguminosarum. J. Gen. Microbiol..

[2] Bybordi A. (2010). The influence of salt stress on seed germination, growth and yield of canola cultivars. Notulae Botan. Hort. Agrobot. Cluj-Napoca.

[3] Ferdous T.A., Kabir S.M.L., Amin M.M., Hossain K.M.M. (2013). Identification and antimicrobial susceptibility of salmonella species isolated from washing and rinsed water of broilers in pluck shops. Int. J. Anim. Vet. Adv..

[4] Goswami D., Dhandhukia P., Patel P., Thakker J.N. (2014). Screening of PGPR from saline desert of Kutch: growth promotion in Arachis hypogea by Bacillus licheniformis A2. Microbiol. Res..

[5] Gull A., Ahmad Lone A., Ul Islam Wani N (2019).

[6] Hashem F.M., Swelim D.M., Kuykendall L.D. (1998). Identification and characterization of salt and thermo-tolerant Leucaena nodulating Rhizobium strains. BiolFertil Soils.

[7] Mahmud F.M.A., Islam M.A., Rubel M.H., Mukharjee S.K., Kumar M., Bhattacharya P., Ahmed F (2023). Effects of halotolerant rhizobacteria on rice seedlings under salinity stress. Sci. Total Environ..

[8] Ji S.H., Gururani M.A., Chun S.C. (2014). Isolation and characterization of plant growth promoting endophytic diazotrophic bacteria from Korean rice cultivars. Microbiol. Res..

[9] Kenasa G., Jida M., Assefa F. (2014). Characterization of phosphate solubilizing faba bean (*Vicia faba* L.) nodulating rhizobia isolated from acidic soils of Wollega. Ethiopia. Sci. Technol, Arts Res. J..

[10] Lindström K., Lehtomaiki S. (1988). Metabolic properties maximum growth temperature and phage sensitivity of Rhizobium sp. (Galega) compared with other fast-growing rhizobia. FEMS Microbiol. Lett..

[11] S. McDevitt, 2009. Methyl Red and Voges-Proskauer Test Protocols. http://www.asmscience.org/content/education/protocol/protocol.3204 (Accessed 18 September 2017).

[12] Rahman S.S., Hossain M.M., Choudhury N. (2013). Effect of various parameters on the growth and ethanol production by yeasts isolated from natural sources. Bangladesh J. Microbiol. vol..

[13] S.S. Rahman, R. Siddique, N. Tabassum, 2017. Isolation and identification of halotolerant soil bacteria from coastal Patenga area. BMC Research Note. 10.1186/s13104-017-2855-7. PMC5663095 29084602

[14] Richardson A.E., Simpson R.J. (2011). Soil microorganisms mediating phosphorus availability update on microbial phosphorus. Plant Physiol..

[15] Rorth M., Jensen P.K. (1967). Determination of catalase activity by means of the Clark oxygen electrode. Biochim. Biophys. Acta.

[16] Singh R.P., Shelke G.M., Kumar A., Jha P.N. (2015). Biochemistry and genetics of ACC deaminase: a weapon to “stress ethylene” produced in plants. Front. Microbiol..

[17] Thompson J.D., Higgins D.G., Gibson T.J. (1994). CLUSTAL W: improving the sensitivity of progressive multiple sequence alignment through sequence weighting, position-specific gap penalties and weight matrix choice. Nucleic. Acids. Res..

[18] Vincent J.M. (1970). International Biology Programme Handbook No. 15.

